# Supplementary material for: MulViMotion: Shape-aware 3D Myocardial Motion Tracking from Multi-View Cardiac MRI
Source: IEEE Trans Med Imaging. Author manuscript; Available in PMC 2022 Aug 3. (PMC7613225; doi:10.1109/TMI.2022.3154599)
Supplement: Supplementary file 1 [file EMS143763-supplement-Appendix.pdf]

# Appendix

## A. Examples of 3D masks

Fig. 12 shows the examples of 3D masks used in the shape regularization module of MulViMotion. These 3D masks identify the locations of multi-view images in the SAX stack. We generate these 3D masks in image preprocessing step by a coordinate transformation using DICOM image header information.

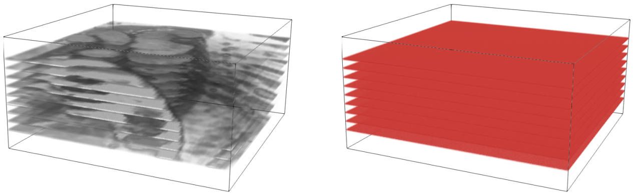

(a) SAX view

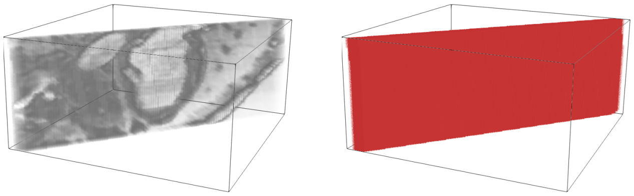

(b) 2CH view

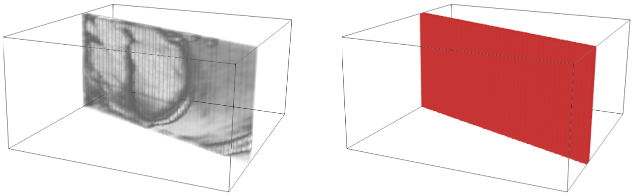

(c) 4CH view

Fig. 12: Examples of 3D masks used in the shape regularization module of MulViMotion. The top row show the 2D images from different anatomical views in the space of the SAX stack. The bottom row show the 3D masks which represent the locations of these 2D images in the SAX stack. (a) The 2D images from SAX view (9 slices). (b) The single 2D image from 2CH view. (c) The single 2D image from 4CH view.

## B. The dynamic videos of motion tracking results

The dynamic videos of motion tracking results of different motion estimation methods have been attached as "Dynamic_videos.zip" in the supplementary material. This file contains four MPEG-4 movies where "FFD.mp4", "dDemons.mp4", "3D-UNet.mp4" are the results of the corresponding baseline methods and "MulViMotion.mp4" is the result of the proposed method. All methods are applied on the same test subject. The Codecs of these videos is H.264. We have opened the uploaded videos in computers with (1) Win10 operating system, Movies&TV player, (2) Linux Ubuntu 20.04 operating system, Videos player, and (3) Mac OS, QuickTime Player. However, if there is any difficulty to open the attached videos, the same dynamic videos can be found in *https://github.com/qmeng99/dynamic_videos/blob/main/README.md*

## C. Additional 3D motion tracking results

Fig. 13 shows the additional 3D motion tracking results on a test subject with slice misalignment. This test subject is the same subject used in Fig. 10 in the main paper. These more results further demonstrate that the proposed method is able to reduce the negative effect of slice misalignment on 3D motion tracking. In addition, we have computed more established clinical biomarkers. Fig. 14 shows the temporal ejection fraction across the cardiac cycle.

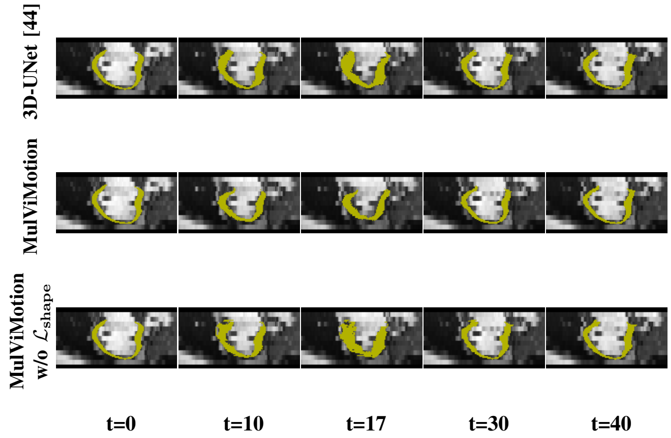


(a) Warped 3D segmentation overlaid on SAX view

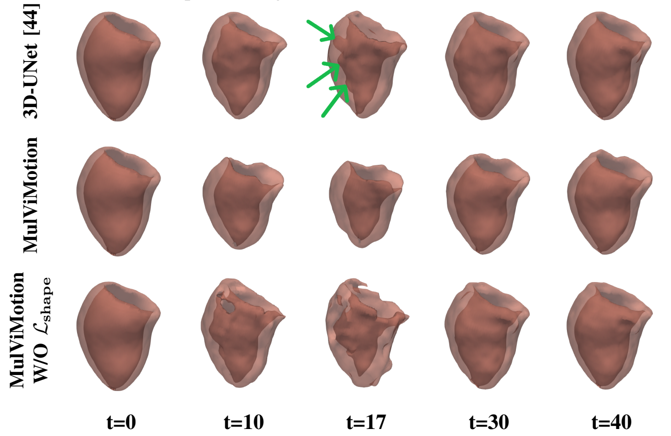


(b) 3D visualization

Fig. 13: Motion tracking results on the test subject with slice misalignment. using 3D-UNet [44], MulViMotion, and MulViMotion without $\mathcal{L}_{shape}$. (a) The warped 3D segmentation overlaid on SAX view. (b) The 3D visualization of the motion tracking results. The green arrows show examples of motion tracking failures using 3D-UNet. Note that we show results in frame $t = 17$ for a more distinct comparison.

## D. Applications

*1) Strain estimation:* Myocardial strain provide a quantitative evaluation for the total deformation of a region of tissue during the heartbeat. It is typically evaluated along three orthogonal directions, namely radial, circumferential and longitudinal. Here, we evaluate the performance

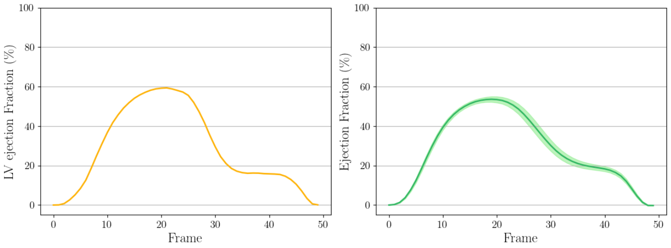


(a) A single test subject (b) All test subjects

Fig. 14: The results of temporal ejection fraction across the cardiac cycle. (a) Results on a randomly selected test subject. (b) Results on all test subjects (mean values and confidence interval are presented).

of the proposed method by estimating the three strains based on the estimated 3D motion field $\Phi_t$. The myocardial mesh at the ED frame is warped to the $t$-th frame using a numeric method and vertex-wise strain is calculated using the Lagrangian strain tensor formula [55] (implemented by https://github.com/Marjola89/3Dstrain_analysis). Subsequently, global strain is computed by averaging across all the vertices of the myocardial wall.

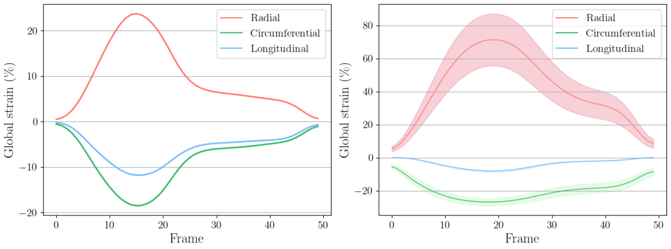


(a) Single subject (b) All test subject

Fig. 15: Global strains across the cardiac cycle which are estimated base on MulViMotion. (a) Results on a randomly selected test subject. (b) Results on all test subjects (mean values and confidence interval are presented).

Fig. 15 shows the estimated global strain curves on test subjects. Both the shapes of the curves and the value ranges of peak strains are consistent with reported results in the literature [52], [56], [57], *i.e.*, radial peak strain is $\sim 20\%$ to $\sim 70\%$, circumferential peak strain is $\sim -15\%$ to $\sim -22\%$ and longitudinal peak strain is $\sim -8\%$ to $\sim -20\%$.

To get more reference strains, we have separately computed global longitudinal and circumferential strains on the 2D LAX and SAX slices according to the algorithm in [14]. On the test set, global longitudinal peak strain is $-18.55\% \pm 2.74\%$ (ours is $-9.72\% \pm 2.49\%$) while global circumferential peak strain is $-22.76\% \pm 3.31\%$ (ours is $-27.38\% \pm 9.63\%$). It is possible that our strains are different from these strains. This is because these strains in [14] are computed only on sparse 2D slices by 2D motion field estimation, and in contrast, we compute global strains by considering the whole myocardium wall with 3D motion fields.

Compared to echocardiograpy, another widely used imaging modality for strain estimation, the average circumferential peak strain reported in our work ($-27.38\%$) is consistent with those typically reported in echocardiograpy ($\sim -22\%$ to $\sim -32\%$ [58]). The average longitudinal peak strain in our study ($-9.72\%$) is lower than that reported in echocardiograpy ($\sim -20\%$ to $\sim -25\%$ [58]). This difference is likely due to the higher spatial and temporal resolution of echocardiography (*e.g.*, $0.2-0.3mm$ for spatial resolution and $40-60$ frames/s for temporal resolution) compared to CMR (*e.g.*, our data has $\sim 1.8mm$ in-plane resolution, $\sim 10mm$ through-plane resolution and 50 frames/heart-beat temporal resolution) [41], [58].

For strain estimation, our results are in general consistent with the value ranges reported in [52], [56], [57]. However, it has to be noted that we calculate the strain based on 3D motion fields, whereas most existing strain analysis methods or software packages are based on 2D motion fields, i.e. only accounting for in-plane motion within SAX or LAX views. This may result in difference between our estimated strain values and the reported strain values in literature. In addition, there is still a lack of agreement of strain value ranges (in particular for radial strains) even among mainstream commercial software packages [57]. This is because strain value ranges can vary depending on the vendors, imaging modalities, image quality and motion estimation techniques [57], [58]. It still requires further investigations to set up a reference standard for strain evaluation and to carry out clinical association studies using the reported strain values. Moreover, when manual segmentation is available, it could be used to provide more perfect and accurate shape constraint, which may further improve 3D motion estimation and thus strain estimation.
